# Research integrity and data ethics in AI-driven integrated healthcare: a critical appraisal

**DOI:** 10.3389/fpubh.2026.1838551

**Published:** 2026-05-28

**Authors:** Wei Zeng, Hong Wei, Yongkai Chen, Jing Tang, Jingyi Ge, Huilong Chen

**Affiliations:** 1 Department of Central Sterile Supply, The First People's Hospital of Yuhang District, Hangzhou, Zhejiang, China; 2 Department of Medical Record, Sanming First Hospital Affiliated to Fujian Medical University, Sanming, Fujian, China; 3 Institute of Medicine Nursing, Hubei University of Medicine, Shiyan, Hubei, China; 4 Department of Information, Sanming First Hospital Affiliated to Fujian Medical University, Sanming, Fujian, China

**Keywords:** algorithmic bias, artificial intelligence, data ethics, governance framework, integrated healthcare, research integrity

## Abstract

The convergence of artificial intelligence (AI) and healthcare is reshaping clinical practice, yet this transformation raises pressing questions about scientific rigor and ethical responsibility. This review provides a critical appraisal of research integrity and data ethics considerations specific to AI implementation in integrated healthcare settings. We analyzed peer-reviewed literature from 2019 to 2025, focusing on algorithmic transparency, model validation and reproducibility, bias detection, privacy protection, informed consent paradigms, and governance frameworks. Our analysis reveals a fundamental tension: the data-intensive nature of AI development often conflicts with established principles of patient autonomy and data protection. The opacity of deep learning models challenges conventional standards of scientific transparency, while datasets reflecting historical healthcare disparities risk encoding and amplifying bias. We propose an integrated governance model that aligns technical validation with ethical oversight, emphasizing the need for prospective clinical trials, diverse stakeholder engagement, and adaptive regulatory approaches. This review offers practical guidance for researchers, clinicians, and policymakers navigating the complex intersection of AI innovation and healthcare ethics.

## Introduction

Artificial intelligence (AI)—encompassing machine learning algorithms, deep neural networks, natural language processing, and computer vision systems—has entered virtually every domain of modern medicine, from interpreting medical images to predicting disease trajectories and personalizing treatment regimens (1, 2). Recent developments, including large-scale foundation models and multimodal systems capable of integrating imaging, genomic, and clinical data simultaneously, have substantially expanded these capabilities beyond what was conceivable even 5 years ago (3, 4). This technological shift is particularly pronounced in integrated healthcare systems, where coordinated care across providers and settings generates vast repositories of clinical data amenable to computational analysis (5). The scale and complexity of these datasets have enabled AI applications that were inconceivable a decade ago, yet they also expose fundamental vulnerabilities in how we conduct, report, and govern medical research.

Two interrelated imperatives must guide this transformation. First, research integrity—the commitment to transparency, reproducibility, and honest reporting—faces novel challenges when algorithms replace or augment human judgment. Traditional peer review struggles to evaluate studies where key methodological details reside in millions of model parameters rather than explicit analytical steps (6). Second, data ethics—encompassing privacy, consent, and equitable access—acquires new dimensions when patient information fuels iterative algorithm development with uncertain downstream applications (7, 8). Governing this lifecycle demands frameworks that align regulatory requirements with the technical maturity of AI systems, progressively tightening oversight as models move from training through real-world testing to post-marketing monitoring (9).

The intersection of these concerns is neither theoretical nor remote. A widely deployed algorithm for identifying patients requiring additional care was found to systematically underestimate illness severity in Black patients, not because of flawed programming but because historical data reflected existing disparities in healthcare utilization (10). Subsequent empirical investigations have confirmed and extended this finding: deep learning models can recognize patient race from medical images alone—raising concerns about embedded demographic information that algorithms may exploit in ways developers neither intend nor detect (11). Even when racial and ethnic categories are explicitly excluded from training features, granular disparities in diagnostic accuracy persist across subgroups, indicating that bias operates through complex, indirect pathways that aggregate performance metrics conceal (12). Such examples underscore that technical sophistication cannot substitute for ethical vigilance. The present review examines how research integrity and data ethics challenges manifest across the AI development lifecycle, from data collection through clinical deployment. We synthesize recent evidence to identify best practices and propose an integrated framework that addresses both scientific and ethical dimensions of healthcare AI.

## AI applications in contemporary healthcare

**Figure 1:**
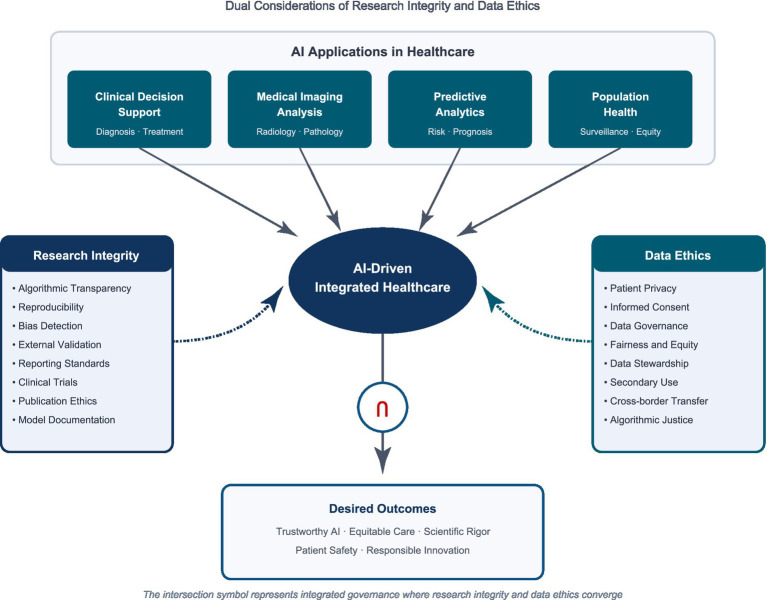
Conceptual framework for AI integration in healthcare with dual ethical imperatives. The framework illustrates how AI applications (clinical decision support, medical imaging analysis, predictive analytics, and population health) operate within the integrated healthcare ecosystem. Research integrity (left) and data ethics (right) represent two complementary pillars that must converge to achieve desired outcomes including trustworthy AI, equitable care, and scientific rigor. Dashed arrows indicate bidirectional influence between ethical considerations and AI implementation.

The past decade has witnessed exponential growth in AI applications across clinical medicine. Bibliometric analyses document a >30-fold increase in healthcare AI publications between 2010 and 2024, with particularly rapid expansion in oncology, cardiology, and radiology (13). This section surveys the major application domains; Figure 1 illustrates the conceptual framework within which these applications operate, with research integrity and data ethics serving as the two pillars governing responsible AI implementation across all domains.

### Clinical decision support

AI-powered clinical decision support systems (CDSS) aggregate information from electronic health records, laboratory values, imaging studies, and genomic data to generate diagnostic and therapeutic recommendations (14, 15). In cardiology, deep neural networks trained on electrocardiogram data have demonstrated cardiologist-level accuracy in detecting arrhythmias (16). Oncology applications extend to recommending personalized chemotherapy regimens informed by tumor genomics (17). These systems hold promise for reducing diagnostic errors and standardizing care quality, particularly in settings where specialist expertise is scarce.

Yet the translation from algorithm development to clinical deployment has proven more difficult than early enthusiasm suggested. Retrospective validation on curated datasets frequently overstates real-world performance (18). When deployed prospectively, many CDSS demonstrate degraded accuracy due to differences in patient populations, clinical workflows, and data collection practices (19). The opacity of complex models—often characterized as “black boxes”—further complicates clinical adoption. Physicians and patients understandably expect explanations for recommendations that influence treatment decisions, creating demand for interpretability that current technical approaches only partially address (20).

### Medical imaging analysis

Computer vision has achieved notable successes in medical image interpretation. Convolutional neural networks detect diabetic retinopathy from fundus photographs with sensitivity comparable to ophthalmologists (21), identify malignant lesions in mammography screening (22), and classify skin cancers from dermatological images (23). These applications address genuine clinical needs, including screening bottlenecks and inter-observer variability among radiologists.

Performance generalization remains a persistent concern. Algorithms trained predominantly on images from well-resourced academic centers may perform poorly when applied to data from community hospitals or low-resource settings with different imaging equipment and protocols (24). Dermatology AI systems trained largely on light-skinned individuals demonstrate reduced accuracy for darker skin tones, illustrating how dataset composition directly shapes clinical utility (25). These limitations underscore the importance of diverse, representative training data and rigorous external validation.

### Predictive analytics and population health

Machine learning models increasingly inform population health management by predicting hospital readmissions, emergency department utilization, and chronic disease progression (26, 27). Such predictions enable proactive interventions, aligning with value-based care models that emphasize prevention over treatment. The COVID-19 pandemic accelerated interest in epidemiological AI applications, with algorithms analyzing mobility patterns and clinical reports to forecast infection spread (28).

Predictive models inherit the biases embedded in their training data. If historical records reflect differential access to care—whether due to insurance status, geography, or discrimination—models may perpetuate these inequities under the guise of objective prediction (29). Risk stratification tools that inadvertently disadvantage vulnerable populations raise ethical concerns that extend beyond technical accuracy to questions of distributive justice. The overarching conceptual framework governing all these applications—including the bidirectional relationship between AI implementation and its dual pillars of research integrity and data ethics—is illustrated in Figure 1.

## Research integrity challenges

### Transparency and explainability

Scientific transparency enables scrutiny, replication, and iterative improvement—principles foundational to evidence-based medicine. Deep learning models with millions of parameters resist straightforward interpretation, creating what critics term the “black box” problem (30). The field of explainable AI (XAI) has responded with a diverse toolkit of methods that span two fundamentally different paradigms: post-hoc explanation techniques that approximate the reasoning of a trained model after the fact, and intrinsically interpretable (“by design”) approaches that constrain model architecture to enable direct inspection (31, 32).

Post-hoc methods dominate current clinical AI implementations. Gradient-based saliency maps and class activation mapping highlight image regions that influenced a prediction, providing visual explanations accessible to clinicians (31). Local Interpretable Model-agnostic Explanations (LIME) and SHapley Additive exPlanations (SHAP) decompose individual predictions into feature-level contributions grounded in game-theoretic principles, and have been applied across clinical domains including mortality prediction, sepsis risk, and oncological decision-making (33). Transformer-based architectures introduce attention mechanisms that indicate which input regions the model weighted most heavily; in ophthalmology, focused attention in vision transformers has been shown to align with clinically relevant retinal structures in a manner interpretable by specialists (34). Counterfactual explanations provide a complementary perspective by identifying the minimal changes to an input that would alter the model’s output, supporting actionable clinical reasoning in critical care settings (35).

Despite this diversity, post-hoc methods share a fundamental limitation: they approximate rather than faithfully represent model reasoning, and their fidelity may degrade as model complexity increases (36). By-design interpretable models—including decision trees, rule-based systems, and monotonic neural networks—sacrifice some predictive performance in exchange for direct mechanistic transparency, and remain the preferred choice for highest-stakes applications where accountability demands cannot be met by post-hoc approximations (32). Regulatory and clinical contexts therefore require thoughtful selection along the interpretability–performance tradeoff, and standardized reporting of which XAI approach was applied, why, and what its known limitations are, should be required by journal editors and regulatory bodies alike.

### Reproducibility and validation

Reproducibility concerns that have affected biomedical research more broadly pose acute challenges for healthcare AI. Systematic reviews reveal that many published AI studies provide insufficient methodological detail for replication (37, 38). Contributing factors include incomplete reporting of data preprocessing steps, proprietary restrictions on model code and training data, and sensitivity to random initialization during training. Privacy constraints that prevent data sharing—while ethically important—further impede independent validation.

Rigorous validation requires multiple complementary approaches. Internal validation using held-out test sets establishes baseline performance, but this approach is susceptible to optimistic bias when the same dataset is used for both model selection and evaluation. Nested cross-validation—in which an outer loop estimates generalization performance while an inner loop performs hyperparameter tuning—provides an unbiased estimate of expected performance and should be considered the minimum standard for single-institution studies where dataset size precludes a separate hold-out set (39). External validation on independent datasets from different institutions tests generalizability (40, 41); temporal validation assesses performance on data collected after model training, addressing dataset shift (42). Structured guidance for both development and external validation of clinical prediction models now exists (41, 43) and should be routinely consulted by authors and reviewers. Prospective clinical trials remain the gold standard for demonstrating real-world effectiveness, though relatively few AI applications have undergone such evaluation (44).

### Algorithmic bias

Bias enters AI systems through multiple pathways. Training data may underrepresent certain populations, leading to models that perform differentially across demographic groups (45). A landmark empirical study demonstrated that deep learning models trained on medical imaging could identify patient race from chest radiographs with high accuracy—a capability neither programmed nor anticipated by developers—raising profound concerns about how demographic information is embedded in imaging data and may be inadvertently leveraged by algorithms in ways that produce disparate clinical outcomes (11). Granular analyses further reveal that coarse racial and ethnic labeling masks important subgroup disparities: models may appear to perform acceptably by aggregate metrics while systematically underdiagnosing specific communities (12). Historical patterns of discrimination encoded in medical records—such as differential referral rates or diagnostic delays—can be learned and amplified by algorithms (46). The consequences extend beyond statistical disparities to tangible clinical harms when predictions systematically disadvantage already marginalized patients.

Bias detection requires disaggregated performance evaluation across relevant subgroups defined by race, sex, age, socioeconomic status, and geographic region (47). Multiple fairness metrics have been proposed, though mathematical constraints prevent simultaneously satisfying all definitions of fairness (48). Mitigation strategies span the development pipeline: data augmentation and reweighting during preprocessing, fairness constraints during model training, and output calibration during post-processing (49). Technical interventions alone prove insufficient without diverse development teams, community engagement, and ongoing monitoring after deployment.

### Reporting standards

Inadequate reporting undermines the ability of readers, reviewers, and clinicians to critically appraise AI research. Studies frequently omit details essential for replication, such as hyperparameter settings, data splitting procedures, and preprocessing steps (50). Recognition of these deficiencies has prompted development of AI-specific reporting guidelines: CONSORT-AI for randomized trials of AI interventions, SPIRIT-AI for trial protocols, and TRIPOD-AI for prediction model studies (51–53). Adoption remains voluntary and inconsistent, limiting their impact on publication practice.

## Data ethics considerations

### Privacy and data protection

Healthcare AI development requires extensive patient data, creating inherent tension with privacy principles. Electronic health records contain sensitive information whose aggregation and computational analysis generate privacy risks exceeding those of traditional clinical uses (54). Research demonstrating re-identification from ostensibly anonymized datasets challenges assumptions about adequate de-identification, particularly as linkage attacks become more sophisticated (55).

Technical approaches offer partial solutions. Differential privacy adds calibrated noise to datasets, providing mathematical guarantees against individual identification while preserving aggregate statistical properties (56). Federated learning enables model training across decentralized data repositories without centralizing raw patient information (57). Homomorphic encryption permits computation on encrypted data (58). Each technique involves tradeoffs between privacy protection and model performance, and practical implementations face computational overhead and potential vulnerabilities that warrant continued research.

Regulatory frameworks establish baseline protections with substantial jurisdictional variation. The European Union’s General Data Protection Regulation (GDPR) imposes stringent requirements for personal data processing, including a “right to explanation” for automated decisions (59). The United States Health Insurance Portability and Accountability Act (HIPAA) defines protected health information and establishes privacy and security standards (60). The European Union’s AI Act (Regulation EU 2024/1689), the world’s first comprehensive legal framework specifically targeting AI systems, classifies healthcare AI as high-risk and mandates conformity assessments, technical documentation, transparency obligations, and human oversight provisions—establishing clearer but more demanding regulatory pathways for developers seeking access to EU markets (61). Navigating these heterogeneous and rapidly evolving requirements presents considerable challenges for multinational research collaborations and global AI development, necessitating proactive regulatory intelligence as an institutional competency (62, 63) (Table 1).

**Table 1:** Evolution of AI governance: key regulatory and policy milestones alongside corresponding technical developments in healthcare AI (2016–2025).

Year	Regulatory/policy milestone	Corresponding technical development
2016	EU GDPR adopted (effective 2018)	Deep learning surpasses human performance in image classification
2017	FDA initiates AI/ML Software as a Medical Device (SaMD) discussion framework	Dermatologist-level skin cancer classification demonstrated (23)
2018	HIPAA enforcement guidance updated for cloud computing	EHR-based deep learning for clinical prediction at scale (27)
2019	WHO Global Strategy on Digital Health published	First evidence of systematic racial bias in a widely deployed clinical AI algorithm (10)
2020	WHO Ethics and Governance of AI for Health consultation begins	CONSORT-AI, SPIRIT-AI, and TRIPOD-AI reporting guidelines published (39–41)
2021	FDA AI/ML Action Plan for SaMD; WHO AI Ethics Guidance published (83)	Federated learning for medical imaging demonstrated at multi-institutional scale (57)
2022	EU AI Act proposed; healthcare AI designated high-risk category	AI recognition of patient race from medical images documented (11)
2023	G7 Hiroshima AI Process; UN AI governance resolution adopted	Large language models deployed across clinical NLP tasks (90)
2024	EU AI Act (Regulation EU 2024/1689) formally adopted; FDA updates change control guidance	Independent external validation systematized for mammography AI (80); LLM demographic disparities quantified (91)
2025	EU AI Act high-risk provisions enter phased enforcement	General governing frameworks for AI/ML medical devices proposed (62)

### Informed consent

Traditional informed consent assumes that data uses can be specified at collection time. AI development disrupts this assumption through iterative, evolving applications that cannot be fully anticipated (64). A patient consenting to research on diabetes management may not foresee that their data could contribute to training algorithms for unrelated conditions or commercial products. The technical complexity of AI systems further strains meaningful consent, as patients may lack the expertise to understand how their information will be processed.

Alternative consent models attempt to address these limitations. Broad consent permits use for unspecified future research within general categories, sacrificing specificity for practicality (65). Dynamic consent employs digital platforms enabling patients to update preferences as new uses emerge (66). Tiered consent offers graduated options with different levels of data sharing (67). None fully resolves the tension between comprehensive disclosure and practical feasibility, suggesting that consent alone cannot bear the full weight of ethical data governance.

### Fairness and equity

AI systems interact with longstanding patterns of healthcare inequity in complex ways. The potential for technology to reduce disparities—through improved access, standardized care protocols, and objective decision support—coexists with risks of automation that amplifies existing biases (68, 69). Datasets reflecting historical injustices encode these patterns, and models trained on such data may perpetuate discrimination at computational scale.

Multiple dimensions of fairness require consideration. Distributive fairness asks who benefits from AI technologies and whether advantages accrue equitably across populations (70). Procedural fairness examines whether development processes meaningfully include diverse stakeholders (71). Recognition fairness considers whether systems respect human dignity and acknowledge social determinants of health (72). Achieving equity requires explicit attention throughout the AI lifecycle, from problem formulation through deployment and monitoring, informed by engagement with affected communities rather than imposed from above.

### Data governance frameworks

Robust governance structures provide essential infrastructure for ethical AI development. Effective governance encompasses policies and procedures guiding data collection, management, sharing, and use while balancing competing values including innovation, privacy, equity, and accountability (73). Data stewardship emphasizes fiduciary obligations to act in patients’ best interests, including maintaining confidentiality, ensuring data quality, promoting beneficial uses, and advancing health equity (74).

Multi-stakeholder governance models incorporate perspectives from patients, clinicians, researchers, administrators, and community representatives (75). Data access mechanisms such as research data networks and secure enclaves enable analysis without unrestricted distribution, balancing openness with protection (76). Real-world experience with regional imaging biobanks illustrates the practical governance complexities involved. The NAVIGATOR initiative, an Italian regional imaging biobank for precision oncology, has systematically documented the legal, ethical, and interoperability challenges encountered when aggregating multi-institutional imaging data for AI model development, demonstrating that governance must address not only data access policies but also metadata standardization, consent harmonization across institutions, and the sustained engagement of clinical stakeholders (77). Its subsequent expansion to a multimodal AI-powered platform integrating imaging, pathological, and molecular data has further underscored how governance complexity scales non-linearly with data modality breadth (78). Analogous challenges have been documented in large-scale multicenter machine learning studies for prostate cancer detection, where governance provisions for data provenance, site-specific calibration, and independent validation proved as demanding as the technical model development itself (79). The distributed nature of integrated healthcare systems—spanning multiple institutions and jurisdictions—complicates governance, requiring federated approaches that maintain alignment on core principles while accommodating local variation.

## Toward integrated governance

Research integrity and data ethics represent complementary rather than competing imperatives. Transparent reporting practices enhance reproducibility while enabling ethical scrutiny. Diverse, representative datasets improve model generalizability and reduce discriminatory bias. Privacy-preserving techniques that enable broader data access can accelerate validation studies. An integrated approach recognizes these synergies while acknowledging genuine tensions that require deliberate navigation.

Translating this integrated vision into practice confronts concrete obstacles that governance frameworks must address directly. Real-world deployment programs have extensively documented that even rigorously developed algorithms degrade when applied in clinical environments different from their training context—driven by heterogeneous imaging equipment, differing clinical workflows, and shifting patient demographics (80). Regional imaging biobank initiatives face persistent tension between data sharing imperatives and privacy protection mandates, and real-world experience confirms that governance must actively manage—rather than assume away—the operational limits of such initiatives (77, 81). Federated learning architectures offer a partial solution by enabling model training across decentralized repositories without centralizing raw patient data, yet real-world implementations reveal governance challenges around data heterogeneity across sites, parameter management for shared model weights, and quality verification without direct data access (82). Multimodal AI systems amplify these challenges further: governance must ensure that independent validation sites possess comparable multi-modal data resources and that site-specific calibration procedures do not introduce new sources of bias (78, 79). These experiences collectively underscore that governance frameworks must be designed for realistic implementation conditions rather than idealized assumptions.

**Figure 2:**
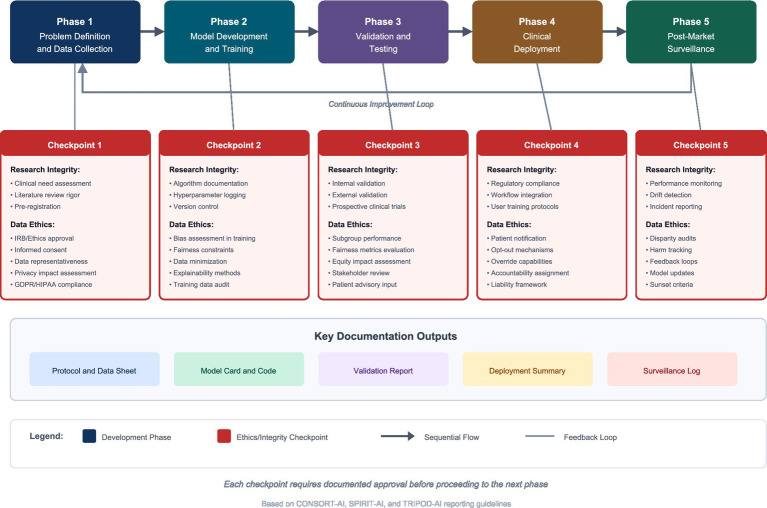
AI development lifecycle with embedded research integrity and data ethics checkpoints. Each development phase (problem definition, model development, validation, deployment, and post-market surveillance) requires documented approval at corresponding checkpoints before progression. Research integrity requirements (upper section of each checkpoint) and data ethics considerations (lower section) are evaluated in parallel. The feedback loop enables continuous improvement based on real-world performance monitoring. Key documentation outputs are specified for each phase to ensure transparency and reproducibility.

Institutional implementation requires dedicated AI oversight structures with authority to review proposed applications, mandate validation standards, and monitor deployed systems (83, 84). Governance committees should include diverse expertise spanning clinical medicine, data science, ethics, law, and patient advocacy. Policies should establish clear procedures for bias assessment and equity impact analysis prior to deployment, with ongoing surveillance for performance degradation or emergent harms. The lifecycle governance model proposed in this review (Figure 2) operationalizes these principles through five sequential phases, each requiring documented checkpoint approval before progression. (1) Problem Definition: the clinical use case, target patient population, potential equity impacts, and key performance requirements are formally documented, with equity stakeholder consultation mandated before development begins. (2) Model Development: training data provenance, demographic composition, class balance, and preprocessing procedures are specified in a model card; an initial bias audit is completed before model freeze. (3) Validation: internal validation on held-out test sets (or nested cross-validation where dataset size requires it) is supplemented by external validation on demographically diverse datasets from independent institutions, with performance reported disaggregated by race, sex, age, and socioeconomic status; data sharing constraints that preclude full external validation must be documented and approved by the governance committee as a known limitation. (4) Deployment: monitoring protocols, alert thresholds for performance degradation, and escalation procedures are activated; clinician-facing explanations are provided for algorithmic recommendations using the most appropriate XAI method for the clinical context. (5) Post-Market Surveillance: longitudinal performance tracking with predetermined triggers for re-evaluation or model withdrawal, including systematic bias re-assessment as clinical practice patterns evolve. Each phase generates structured documentation outputs that inform upstream revisions and create an auditable governance record.

Workforce development is equally critical. Medical education must integrate AI literacy encompassing technical fundamentals, clinical applications, and ethical considerations (85). Data scientists require exposure to healthcare domain knowledge and ethical frameworks. Interdisciplinary collaboration—bringing together clinicians, engineers, ethicists, and affected communities—enables holistic assessment that no single discipline can provide independently (86).

**Figure 3:**
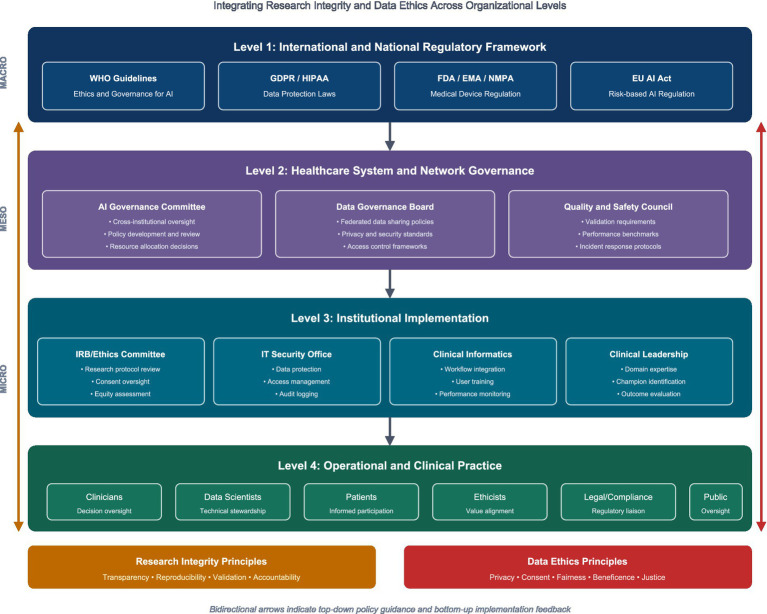
Multi-level governance framework for healthcare AI integrating research integrity and data ethics. The framework operates across four hierarchical levels: (1) international and national regulatory bodies establishing overarching standards; (2) healthcare system governance committees coordinating network-wide policies; (3) institutional implementation through ethics committees, IT security, clinical informatics, and clinical leadership; and (4) operational stakeholders including clinicians, data scientists, patients, and ethicists. Bidirectional arrows indicate top-down policy guidance and bottom-up implementation feedback. Research integrity principles (transparency, reproducibility, validation, accountability) and data ethics principles (privacy, consent, fairness, beneficence, justice) underpin all governance levels.

Regulatory approaches must balance enabling innovation with protecting patients. Risk-based frameworks calibrate oversight intensity to potential harms, applying greater scrutiny to high-stakes diagnostic applications than low-risk administrative tools (87, 88). The EU AI Act’s high-risk designation for healthcare AI formalizes this principle at the legislative level, requiring conformity assessments, technical documentation, human oversight provisions, and post-market monitoring plans aligned with the governance lifecycle described above (9, 61). Adaptive regulation permits updating requirements as evidence accumulates and technologies evolve. International harmonization reduces fragmentation while respecting jurisdictional differences in values and legal traditions (89). The multi-level governance framework illustrated in Figure 3 translates regulatory intent into operational practice across four hierarchical levels. At the apex, international regulatory bodies and national agencies establish overarching standards. Healthcare system governance committees at the second level translate these standards into network-wide policies accommodating institutional variation while maintaining alignment on core principles. At the institutional level, ethics committees, IT security teams, clinical informatics departments, and clinical leadership jointly implement policies. At the operational level, the framework explicitly incorporates clinicians, data scientists, patients, and ethicists as active governance participants. The bidirectional architecture is critical: top-down policy guidance is complemented by structured bottom-up implementation feedback, enabling governance to evolve in response to frontline experience. Research integrity principles—transparency, reproducibility, validation, accountability—and data ethics principles—privacy, consent, fairness, beneficence, justice—underpin all four levels.

## Future directions

The healthcare AI landscape continues evolving rapidly. Foundation models and large language models trained on massive datasets demonstrate impressive capabilities across clinical text generation, question answering, and diagnostic reasoning, but raise unresolved questions about training data provenance, consent for secondary use, and potential for generating misinformation (3, 90). Systematic analyses reveal substantial performance disparities across demographic groups in clinical LLM deployments, with models exhibiting reduced accuracy and higher rates of potentially harmful outputs for historically underrepresented populations—reproducing at scale the bias patterns documented in narrower AI systems (91, 92). Frameworks for rigorous clinical evaluation of LLMs remain nascent, and methodological standardization represents an urgent research priority (93). Multimodal systems integrating imaging, genomic, and clinical data promise comprehensive patient modeling but amplify both privacy risks and validation complexity (4). Continuously learning algorithms that update based on deployment experience present governance challenges, as performance may drift in unpredictable directions (94).

Substantial research gaps persist. Prospective studies evaluating real-world AI implementation outcomes remain scarce relative to retrospective algorithm development (95). Long-term surveillance tracking model performance over years rather than months is virtually absent. Comparative effectiveness research evaluating different AI approaches against each other and against conventional care would inform evidence-based adoption (96). Methodological innovation is needed for clinical trial designs that accommodate adaptive algorithms, fairness assessment frameworks that acknowledge competing definitions, and privacy techniques that preserve clinical utility (97).

## Conclusion

Artificial intelligence is transforming healthcare in ways both promising and perilous. Realizing the benefits while mitigating harms requires simultaneous attention to research integrity—encompassing transparency, reproducibility, and bias awareness—and data ethics—including privacy, consent, and equity. These imperatives are not obstacles to innovation but foundations enabling trustworthy, sustainable AI integration.

The path forward demands institutional investment in governance structures and workforce capacity, regulatory frameworks that balance protection with flexibility, and sustained engagement with patients and communities whose data and lives are at stake. Technical excellence must be accompanied by ethical reflection, rigorous validation, and genuine stakeholder participation. By maintaining commitment to both scientific rigor and ethical responsibility, we can guide AI’s integration into healthcare toward outcomes that serve all patients equitably.
